# The influence of single nucleotide polymorphisms on the association between dietary acrylamide intake and endometrial cancer risk

**DOI:** 10.1038/srep34902

**Published:** 2016-10-07

**Authors:** Janneke G. F. Hogervorst, Piet A. van den Brandt, Roger W. L. Godschalk, Frederik-Jan van Schooten, Leo J. Schouten

**Affiliations:** 1 Centre for Environmental Sciences, Hasselt University, Diepenbeek, Belgium; 2 Department of Epidemiology, School for Oncology & Developmental Biology (GROW), Maastricht University, Maastricht, the Netherlands; 3 Department of Pharmacology and Toxicology, School for Nutrition and Translational Research in Metabolism (NUTRIM), Maastricht University, Maastricht, the Netherlands

## Abstract

It is unclear whether the association between dietary acrylamide intake and endometrial cancer risk as observed in some epidemiological studies reflects a causal relationship. We aimed at clarifying the causality by analyzing acrylamide-gene interactions for endometrial cancer risk. The prospective Netherlands Cohort Study on diet and cancer includes 62,573 women, aged 55–69 years. At baseline, a random subcohort of 2589 women was selected for a case cohort analysis approach. Acrylamide intake of subcohort members and endometrial cancer cases (n = 315) was assessed with a food frequency questionnaire. Single nucleotide polymorphisms (SNPs) in genes in acrylamide metabolism, sex steroid systems, oxidative stress and DNA repair were assessed through a MassARRAY iPLEX Platform. Interaction between acrylamide and SNPs was assessed with Cox proportional hazards analysis, based on 11.3 years of follow-up. Among the results for 57 SNPs and 2 gene deletions, there were no statistically significant interactions after adjustment for multiple testing. However, there were nominally statistically significant interactions for SNPs in acrylamide-metabolizing enzymes: CYP2E1 (rs915906 and rs2480258) and the deletions of *GSTM1* and *GSTT1*. Although in need of confirmation, the interactions between acrylamide intake and CYP2E1 SNPs contribute to the evidence for a causal relationship between acrylamide and endometrial cancer risk.

Acrylamide is a probable human carcinogen (IARC class 2A; based on rodent studies) that was discovered in 2002 in various heat-treated carbohydrate-rich foods, such as cookies, potato chips, French fries and coffee. Since then, epidemiological studies have been performed in order to investigate the impact of dietary acrylamide intake on human cancer risks. The results of these studies are inconsistent: for some cancers (endometrial, ovarian, breast and kidney cancer) increased risks have been observed in some studies but not all. A recent meta-analysis on the association between acrylamide intake and endometrial cancer risk shows a pooled relative risk for high vs. low intake of 1.39 (95% CI 1.09–1.77) in never-smokers^1^. In the most recent risk assessment of acrylamide, by the European Food Safety Authority (EFSA)^2^, the epidemiological findings on acrylamide and cancer risk are discussed but not incorporated in the actual risk assessment. The most important reason for this is that the causality of the observed associations is still unclear, mainly due to the inconsistent associations across studies.

It is important to get more clarity on the causality of the observed epidemiological associations. They indicate that risks may be present at current dietary intake levels and they are still an underestimation of the true risk because of random measurement error of the acrylamide intake. In addition, the observed risks are considerably higher than predicted from rodent studies^3^. Moreover, virtually everyone is exposed to acrylamide through diet.

It is generally thought that acrylamide may cause cancer through the genotoxic action of acrylamide’s metabolite glycidamide (generated by the action of cytochrome P4502E1 (CYP2E1)) but other mechanisms, such as effects on sex hormones, are hypothesized as well^2^.

In the present study, we investigated whether genetic make-up modifies the association between dietary acrylamide intake as assessed through a validated food frequency questionnaire and endometrial cancer risk, thereby contributing to evidence on acrylamide’s mechanism of action and the causality of the observed associations between acrylamide and endometrial cancer risk. We selected SNPs in candidate genes involved in acrylamide metabolism (*CYP2E1, glutathione-s-transferases*, and *epoxide hydrolase*) and in mechanisms through which acrylamide is hypothesized to cause cancer: mechanisms involving DNA damage, sex hormones, and oxidative stress^4^. We specifically also look at never-smokers because cigarette smoke is an important source of acrylamide exposure^1^.

## Results

Table 1 shows the characteristics of the subcohort and endometrial cancer cases at baseline. Cases were more often never-smokers, smoked fewer cigarettes per day and for a shorter duration. They more often ever used postmenopausal hormone treatment and considerably less often ever used oral contraceptives. Cases had a lower age at menarche and a later age at menopause and they had fewer children. In addition, cases had a considerably higher BMI and more often a family history of endometrial cancer.

### Main effect of acrylamide

There was no main effect of acrylamide with 20.3 years of follow-up (HR of highest versus the lowest quintile of intake: 1.03 (95% CI 0.71–1.51) and 0.98 (0.88–1.10) per 10 μg/day increment of intake (Table 2). A similar null association was observed for never-smokers. We decided to further focus on acrylamide-gene interactions for the first 11.3 years of follow-up period, for which we did see an association between acrylamide intake and endometrial cancer risk^5^.

### Main effect of SNPs

Table 3 lists the SNPs that showed a (borderline) statistically significant association with endometrial cancer risk (11.3 years of follow-up). Women with variant alleles of rs1056827 in *CYP1B1*, rs944722 in *NOS2*, and rs2228000 in *XPC* showed a decrease in risk (p-trend 0.04, 0.05, 0.06, respectively). Women with variant alleles of rs2472299 in *CYP1A2*, rs3219489 in *MUTYH*, rs660149 in *PGR*, and rs1042157 and rs6839 in *SULT1A1* showed a positive trend over the number of variant alleles (p-trend 0.05, 0.09, 0.05, 0.07 and 0.07, respectively). A decreased risk of endometrial cancer was observed in women with a homozygous deletion of the *GSTM1* gene when both SNP selected to represent the deletion were not called: HR: 0.80 (0.58–1.11). The association was similar when the deletion was based on missing calls in rs10857795 alone and there was a statistically significant decrease in endometrial cancer risk when the deletion was based on rs200184852 alone (HR 0.71 (0.52–0.96)). However, none of the SNPs was statistically significantly associated with endometrial cancer risk after adjustment for multiple comparisons; none of the Benjamini-Hochberg FDR values were lower than the chosen 0.20 threshold.

### Interactions between acrylamide intake and SNPs

None of the SNPs showed a statistically significant multiplicative interaction with acrylamide for the 11.3 year follow-up period when adjusted for multiple comparisons. In Table 4, we show interactions with SNPs in genes involved in acrylamide metabolism that are interesting because they have a higher *a priori* probability of modifying the association between acrylamide and cancer risk than the other selected SNPs because they determine internal exposure to acrylamide and glycidamide.

We observed nominally (borderline) statistically significant multiplicative interaction for 2 SNPs in *CYP2E1*: rs915906 for all women (p-interaction = 0.02) and for never-smoking women (p-interaction = 0.07), and rs2480258 for all women (p-interaction = 0.03). There only was an association between acrylamide and endometrial cancer risk in homozygous wild types for both SNPs.

We observed nominally statistically significant multiplicative interaction for the deletion of *GSTT1* for all women (p-interaction < 0.001) and never-smoking women (p-interaction = 0.02), although based on few cases with a homozygous deletion of *GSTT1* (12 among all women and 7 among never-smoking women). Acrylamide was only positively associated with endometrial cancer risk in women with at least one copy of the *GSTT1* allele. For the *GSTM1* gene deletion, the same pattern of associations was observed: the acrylamide-associated risk of endometrial cancer was only increased in women with at least one copy of the *GSTM1* gene but there was no statistically significant interaction with acrylamide intake. There were no interactions between acrylamide and SNPs in other acrylamide-metabolizing genes (*GSTA5, GSTP1* and *EPHX1*) (results not shown).

There were some (borderline) nominally statistically significant interactions between acrylamide and other SNPs (Supplemental Table 3): rs11252859 in *AKR1C1* (among never-smokers), rs1042157 and rs6839 in *SULT1A1*, rs3736599 in *SULT1E1* (among never-smokers), rs10432782 in *SOD1* (among never-smokers), rs3448 in *GPX1* (among never-smokers), rs1800566 in *NQO1* (among never-smokers), and rs2472299 in *CYP1A2* (among never-smokers). In addition, differences in the acrylamide dose-response relationship between the genotypes were observed for rs5275 in *PTGS2*, rs1280350 in *MGC12965*, rs1056836 in *CYP1B1*, rs2228000 in *XPC*, rs4986938 in *ESR2*, rs6428830 in *HSD3B1/B2* (among never-smokers) and rs64759180 in *RRM2* (Supplemental Table 3).

## Discussion

The current study is the first to analyze acrylamide-gene interactions for (endometrial) cancer risk. We followed a candidate gene approach for identifying SNPs in genes involved in acrylamide metabolism and genes involved in the mechanisms by which acrylamide might cause cancer: a sex hormonal effect, oxidative stress and DNA damage.

The positive association between acrylamide intake and endometrial cancer risk that we observed previously after 11.3 years of follow-up^5^ was not present after 20.3 years of follow-up. A possible explanation for this is that the positive association observed in the first follow-up period was a spurious finding, making the current acrylamide-gene interaction analysis all the more relevant. Another possible explanation is that the baseline assessment of dietary acrylamide in 1986 is insufficiently representative of the dietary acrylamide intake in the etiologically relevant exposure period for endometrial cancers occurring in the latter half of the follow-up period. For this reason, we focused on the interaction between acrylamide and SNPs in the first 11.3 years of follow-up.

Although there were several SNPs showing a statistically significant interaction with acrylamide intake, none withstood the adjustment for multiple comparisons. However, we observed some nominally statistically significant interactions with SNPs in genes involved in acrylamide metabolism, thus having a higher *a priori* probability of modifying the association between acrylamide and cancer risk than the other selected SNPs.

Glycidamide (formed by epoxidation of acrylamide through CYP2E1) is often thought to be the compound responsible for acrylamide-induced carcinogenesis due to its genotoxicity. Therefore, studying the modifying effect of SNPs in *CYP2E1* on the association between acrylamide and cancer risk contributes important information on the causality of the association. We observed nominally statistically significant multiplicative interaction for 2 SNPs in *CYP2E1*: rs915906 and rs2480258. These 2 *CYP2E1* SNPs are in the intronic region of the gene and thus do not affect the protein code, but they may be in linkage disequilibrium with variants that are causative. It was shown that the allelic variants of both genes and specifically their combination were associated with an increase in micronuclei (MN) count in binucleated lymphocytes, a marker of DNA damage and an established risk marker for carcinogenesis^6^. CYP2E1 metabolizes several other compounds than acrylamide, e.g., ethanol, benzene, nitrosamines, and acetaminophen^7^, and the enzyme bioactivates these compounds and thus increases their MN-forming potential. The observed increase in MN count observed with the variant alleles thus suggests increased CYP2E1 activity of the variant alleles or alleles in linkage disequilibrium with these alleles. This then would suggest that acrylamide itself is the causative compound in endometrial carcinogenesis, because the strongest association between acrylamide and endometrial cancer risk was observed in homozygous wild types.

We also studied another SNP in *CYP2E1* (rs6413432), which did not show a statistically significant interaction with acrylamide intake but among never-smoking women, the risk of endometrial cancer was considerably higher in the homozygous wild types than in women with variant alleles: HRs per 10 μg/day increment in acrylamide intake were 1.25 (95% CI: 1.04–1.50, n = 95) and 1.06 (95% CI: 0.61–1.82, n = 25) for homozygous wild types versus women with variant alleles, respectively. This result points in the same hypothesized direction of acrylamide itself being the more relevant compound as the result for the other 2 SNPs because the variant allele of rs6413432 leads to increased *CYP2E1* gene expression^8^ and the association between acrylamide and endometrial cancer risk was strongest in women homozygous for the wild type allele.

The observed interaction with these *CYP2E1* SNPs contributes to the evidence for a causal association between acrylamide and endometrial cancer risk. Acrylamide has a high affinity for binding to thiol groups in proteins. Its effect on the nervous system is hypothesized to occur through binding to and disruption of proteins involved in neurotransmission^9^. For neurotoxicity, it is hypothesized that acrylamide itself is mainly responsible because acrylamide has a higher affinity for binding to proteins than glycidamide^10^. Despite the fact that a lot of attention is given to the genotoxicity of acrylamide’s metabolite glycidamide as the mechanism of action, it is also hypothesized that acrylamide causes cancer through other mechanisms, such as effects on sex hormones. Those mechanisms may involve disruption of key proteins, in which acrylamide itself could be the causative compound.

We observed that women with at least one copy of *GSTM1* and *GSTT1* were at an increased acrylamide-associated risk of endometrial cancer, which was contrary to what we expected. Both acrylamide and glycidamide are detoxified by conjugation to glutathione and urinary excretion of the mercapturic acid complexes^11^. However, it is unclear if glutathione conjugation of acrylamide occurs non-enzymatically or through catalyzation by GSTs^12^. Interestingly, regardless of acrylamide intake, women with a double deletion of *GSTM1* were at a decreased risk of endometrial cancer in our study, which has been observed before^13^, and also for some other cancers^14^,^15^. A possible explanation is that GSTs catalyze the conjugation of reduced glutathione (GSH) to compounds that protect against endometrial cancer or that they bioactivate compounds involved in endometrial carcinogenesis, for instance catechol estrogens^16^. In addition, conjugation of acrylamide with GSH can result in depletion of cellular GSH stores, leading to an altered redox status of the cell. This can affect gene expression directly or through regulating various redox-dependent transcription factors^4^. Considering the fact that acrylamide induces GST activity^17^,^18^, it would be expected that the positive association between acrylamide and endometrial cancer is only present among women in whom the activity of GST can be induced; *i.e*. women with at least one copy of the genes.

An interesting observation in this context is that in a study on 85 persons of whom 51 were occupationally exposed to acrylamide, persons with the *GSTM1* null genotype had lower urinary levels of the mercapturic acid metabolite of acrylamide in combination with a higher ratio of the glycidamide mercapturic acid metabolite to the acrylamide mercapturic acid metabolite than *GSTM*1 positive persons^19^. The authors speculate that this indicates that in persons with the *GSTM1* null genotype a higher percentage of acrylamide is converted to glycidamide. In combination with the fact that we only observed an association between acrylamide and endometrial cancer risk in women with at least one copy of *GSTM1*, this could, in line with the results for the *CYP2E1* SNPS, suggest that acrylamide itself is the causative compound in endometrial carcinogenesis. Whatever the biological explanation behind the observed interactions with *GSTs*, it is remarkable that *GSTM1* and *GSTT1* show a similar interaction pattern.

There were some (borderline) nominally statistically significant interactions between acrylamide and other SNPs: rs11252859 in *AKR1C1*, rs1042157 and rs6839 in *SULT1A1*, rs3736599 in *SULT1E1*, rs10432782 in *SOD1*, rs3448 in *GPX1*, rs1800566 in *NQO1*, and rs2472299 in *CYP1A2*. In addition, differences in the acrylamide dose-response relationship between the genotypes were observed for rs5275 in *PTGS2*, rs1280350 in *MGC12965*, rs1056836 in *CYP1B1*, rs2228000 in *XPC*, rs4986938 in *ESR2*, rs6428830 in *HSD3B1/B2* and rs64759180 in *RRM2*. For all these SNPs it is even more important that the interaction between acrylamide intake and these SNPs is first corroborated or refuted in other studies in order to be able to judge whether our findings were chance findings or not. Therefore it is premature to elaborately discuss their possible role in acrylamide-induced endometrial carcinogenesis here.

This study has some limitations. Acrylamide intake was only assessed once, at baseline. The association between acrylamide and endometrial cancer risk was only present in the first half of the 20.3 year follow-up period, possibly due to the fact that the single dietary intake measurement was not representative of the relevant exposure of the later cases. Using the full 20.3 year follow-up period for analysis (results not shown), there were some similar nominally statistically significant interactions as with the 11.3 year follow-up period, namely with rs6839 (*SULT1A1*), rs2472299 (*CYP1A2*), and rsrs3448 (*GPX1*). However, the differences between the genotypes were not as clear as with the 11.3 year follow-up period. The other statistically significant interactions that were observed with 11.3 years of follow-up were not statistically significant with 20.3 years of follow-up. With 20.3 years of follow-up, there were some statistically significant interactions that were not observed with 11.3 years of follow-up: rs11252887 (*AKR1C1*) (only women with 1 or 2 variant alleles showed a clear increase in acrylamide-associated endometrial cancer risk), rs28362491 (*NFKB1*) (increased acrylamide-associated risk only in homozygous wild types), rs2228000 (*XPC*) (increased acrylamide-associated risk only in never-smoking homozygous wild types) and rs5275 (*PTGS2*) (increased acrylamide-associated risk only in homozygous wild types). None of the interactions were statistically significant after adjustment for multiple testing.

Rs2480258 in *CYP2E1* that statistically significantly modified the association between acrylamide intake and endometrial cancer risk was not in Hardy-Weinberg equilibrium, although the deviation was minor (p = 0.03) and not statistically significant after adjustment for multiple testing. This may indicate that the genotypes for this SNP were measured with some error. However, there is no reason to assume that this error is different for cases and subcohort members or for different categories of acrylamide intake. Therefore, this potential genotyping error would rather have led to missing a true interaction (if any) than detecting an interaction^20^.

Some of the interactions that we discussed may be chance findings, considering that none of the interactions withstood the adjustment for multiple comparisons. However, finding interactions for multiple SNPs in the same gene for *CYP2E1* decreases the likelihood that they are chance findings, especially when there are clear differences in the dose-response pattern of acrylamide between the genotypes.

Both the homozygous deletion of *GSTM1* and that of *GSTT1* in our population (based on the combination of SNPs selected for these genes) were low (31% and 8%, respectively) compared to the reported prevalence in Caucasian populations (40–60% for *GSTM1* and 10–20% for *GSTT1*). In a PCR study (not shown), we tested some of the samples (n = 33) that showed a discrepancy in the iPLEX assay between rs10857795 and rs200184852 to represent the *GSTM1* deletion and rs4630 and rs1040309 to represent the *GSTT1* deletion (n = 37). All the samples that had no call for rs200184852 (but did have a call for rs10857795) in the iPLEX assay showed absence of a PCR product in the PCR study (results not shown). Only 51% of the samples that had no call for rs4630 (but did have a call for rs1040309) in the iPLEX assay showed absence of a PCR product in the PCR study. Thus, it can be assumed that the percentage of study participants with a deletion of *GSTM1* is closer to 42% (as reflected by rs200184852) than to the 31% reflected by both *GSTM1* SNPs. For *GSTT1*, it cannot be concluded which SNP best represents absence of the deletion but the true percentage is probably somewhere between 11% (rs104003609) and 15% (rs4630). In conclusion, the percentages of the *GST* deletions in this study are within the ranges of published percentages for Caucasian populations.

Strengths of this study are the prospective nature, the complete follow-up, and the fact that we observed a main effect of acrylamide, which may mean that it was assessed reasonably well in this study.

In conclusion, when we adjusted for multiple comparisons, there was no statistically significant interaction between SNPs and acrylamide intake for endometrial cancer risk. However, the nominally statistically significant interaction between acrylamide and SNPs in *CYP2E1* contributes to the evidence of a causal association between acrylamide intake and endometrial cancer risk but confirmation is needed. Based on this study, we recommend prospective cohort studies on acrylamide-gene interactions and for some genes in particular: *CYP2E1* and *GSTs*. These studies are preferably larger than the present study.

## Methods

### Study Cohort, Cases and Follow-up

The Netherlands Cohort Study on diet and cancer began in September 1986 with the inclusion of 62,573 women aged 55–69 years. Data on dietary habits and other risk factors were collected through a self-administered questionnaire at baseline in 1986. In addition, 75% of the participants sent in toenail clippings. Participants gave informed consent by returning the completed questionnaire. The NLCS, using toenail DNA for genotyping, and associated protocols were approved by the review boards of TNO Nutrition and Food Research (Zeist, the Netherlands) and Maastricht University (Maastricht, the Netherlands). All methods were applied according to the approved guidelines.

Following the case-cohort approach, cases were enumerated for the entire cohort, while the accumulated person-time at risk for the full cohort was estimated from a random subcohort of 2589 women. Since baseline, the subcohort has been followed up regularly for vital status information. Incident cases in the full cohort were detected by annual computerized record linkages to the regional cancer registries and the Netherlands Pathology Registry. Further details on design and methods of follow-up are presented elsewhere^21^,^22^,^23^,^24^.

**Figure 1:**
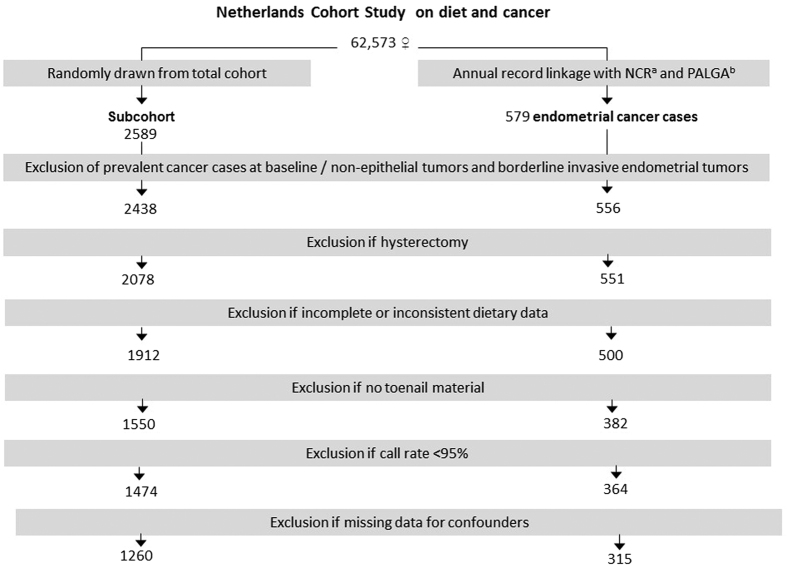
Flow chart of subcohort members and endometrial cancer cases. ^a^NCR = Netherlands Cancer Registry. ^b^PALGA = Dutch Pathology Registry.

After 20.3 years of follow-up (Sept. 1986–Dec. 2006), there were 588 microscopically confirmed primary carcinomas of the endometrium ([ICD-O]-3:C54). Cohort members were excluded from analysis if their dietary data were incomplete or inconsistent, they had not sent in toenail clippings, they had no or inferior (call rate <95%) data on SNPs, or if they reported to have had a hysterectomy. Figure 1 shows the selection and exclusion steps that resulted in the numbers of cases and subcohort members available for analysis.

### Acrylamide Intake Assessment

A food frequency questionnaire with questions on 150 food items was used for estimating dietary habits. The acrylamide intake was estimated from the mean acrylamide level of foods on the Dutch market, and the frequency of consumption and portion size of the foods, as described in detail elsewhere^5^.

### Selection of genes and SNPs

The selection of genes focused on genes involved in 1) acrylamide metabolism (*CYP2E1, GSTs* and *EPHX1*) and 2) the hypothesized mechanisms of acrylamide-induced carcinogenesis:^4^ 2a) a sex hormonal effect (involving sex hormone synthesis/metabolism or sex hormone nuclear receptors); 2b) oxidative stress; 2c) genotoxicity (DNA repair); or 2d) genes, not belonging to 1 or 2a–c, that were shown to be significant in an acrylamide-related polymorphism study^19^,^25^,^26^,^27^,^28^ or because they are in genes that were shown differentially expressed upon acrylamide exposure in acrylamide-related gene expression studies^17^,^18^,^29^,^30^,^31^,^32^,^33^,^34^,^35^,^36^,^37^,^38^,^39^,^40^.

Genes and SNPs of interest were identified from the literature (HugeNavigator and PubMed) and from a personal communication (for SNP rs1280350 in *MGC12965*) with Jos Kleinjans (Dept. of Toxicogenomics, Maastricht University). This latter SNP was shown to be associated with the level of acrylamide-hemoglobin adducts in cord blood of newborns in the Newborns and Genotoxic exposure risks (NewGeneris) project.

Preferably SNPs shown to be associated with a cancer involving sex hormones (endometrial, ovarian, breast and prostate cancer) were selected. However, we also selected some SNPs with no literature on their relation with the cancers of interest but that were shown to be have an association or effect in the above-mentioned acrylamide-related polymorphism study^19^,^25^,^26^,^27^,^28^ or gene expression studies^17^,^18^,^29^,^30^,^31^,^32^,^33^,^34^,^35^,^36^,^37^,^38^,^39^,^40^. It is unsure if *in vitro* or *in vivo* animal gene expression studies can be extrapolated to humans but at least these studies give indications that acrylamide exposure may involve effects on or interfere with these genes/enzymes.

Only validated SNPs with a minor allele frequency ≥10% in dbSNP (Caucasians) were selected. The functionality of the SNPs (as based on the F-value in F-SNP)^41^ and the region of the SNP in the gene were no selection criteria per se but they were used to choose between SNPs when there were many interesting SNPs per gene.

There were too many potentially interesting genes (see Supplemental Table 1), so we prioritized SNPs in acrylamide-metabolizing genes and (SNPs in) genes that showed an association or effect in acrylamide studies on gene polymorphisms and gene expression changes.

*GSTM1* and *GSTT1* are genes that are completely deleted in a large proportion of the population. The beginning and ending of the deleted sequences of *GSTM1* and *GSTT1* are not precisely known. Thus, it was impossible to design 1 assay (based on single base extension) for the deletion, as is done for SNPs. Therefore, we chose 3 SNPs in *GSTM1* and *GSTT1* each to represent the deletions (see Supplemental Table 1); when all 3 SNPs were not called, we assumed deletion of the gene.

66 SNPs were designed to fit together onto the 2 multiplexes that we budgeted: 6 SNPs to determine the *GST* deletions and 60 SNPs in other genes, see Supplemental Table 2.

### DNA isolation and genotyping

DNA was isolated from 15 mg of toenail clippings, following a protocol described elsewhere^42^. Genotyping was performed by Agena, on the MassARRAY platform using the iPLEX TM assay^43^. This method has been used before to successfully genotype DNA from toenails^42^,^44^,^45^.

5% of the samples (n = 190) were duplicate samples to check the reproducibility of genotyping, which was >99%. Supplemental Table 2 shows the 60 SNPs that were analyzed. Three of the 60 SNPs that were genotyped had a call rate <80% and were excluded from the analyses. Six SNPs out of the remaining 57 SNPs did not adhere to Hardy-Weinberg equilibrium (p < 0.05). We excluded samples with a call rate <95% (18 cancer cases, 76 subcohort members). With regard to the SNPs selected to represent the *GSTM1* deletion, rs10857795 was not called in 36%, rs200184852 in 42% and rs74837985 in only 2% of the subcohort. The latter value appears to be due to genotyping error. Therefore, we decided to base the assessment of the deletion of the *GSTM1* gene only on rs10857795 and rs200184852. 31% had a missing value for both rs10857795 and rs200184852. With regard to *GSTT1*, rs2844008 was not called in 58%, rs4630 in 15%, and rs140309 in 11% of the subcohort. 8% had a missing value for all 3 *GSTT1* SNPs.

### Statistical Analysis

Hazard rate ratios (HRs) and 95% confidence intervals were obtained through Cox proportional hazards regression with STATA software (package 13), with standard errors estimated using the robust Huber-White sandwich estimator to account for additional variance introduced by sampling from the cohort. The proportional hazards assumption was tested using scaled Schoenfeld residuals.

Covariables, selected from the literature, for the models of the main effect of acrylamide and acrylamide-gene interactions were: age, body mass index, age at menarche, age at menopause, ever use of oral contraceptives, parity, ever use of postmenopausal hormone, family history of endometrial cancer, and energy intake. Smoking status, the duration of smoking and the number of cigarettes per day were included in the model, because cigarette smoke is an important source of acrylamide. Smokers have been shown to have on average four times higher exposure to acrylamide than non-smokers^46^. Moreover, smoking is inversely associated with endometrial cancer risk^47^. Therefore, subgroup analyses for never-smokers were performed. The main associations between SNPs and endometrial cancer risk were adjusted for age only.

In a previous analysis, we observed a positive main effect of acrylamide intake on endometrial cancer risk after 11.3 years of follow-up^5^. In the present study, our first step was to investigate whether this main effect was also present with 20.3 years of follow-up.

Multiplicative interaction between acrylamide intake and SNPs was tested using product terms of the continuous acrylamide intake variable and genotype. For statistical power reasons, we used a dominant genetic model (i.e., 1 or 2 variant alleles versus homozygous wild type). Tests for acrylamide dose-response trends in genotype strata were performed by fitting the mean acrylamide intake in the tertiles as a continuous variable.

We applied the False Discovery Rate method developed by Benjamini-Hochberg to adjust for multiple testing^48^ with the expected proportion of false positives set at 20%, which is applied regularly in candidate gene studies^49^,^50^. We performed separate adjustment for multiple testing for all women and for never-smoking women.

Two-sided p values are reported throughout this paper.

## Additional Information

**How to cite this article**: Hogervorst, J. G. F. *et al*. The influence of single nucleotide polymorphisms on the association between dietary acrylamide intake and endometrial cancer risk. *Sci. Rep*. **6**, 34902; doi: 10.1038/srep34902 (2016).

## Supplementary Material

Supplementary Information

## Figures and Tables

**Table 1:** Characteristics of subcohort and endometrial cancer cases.

Variable	Endometrial cancer cases	Subcohort
n*	364	1474
*Dietary variables*		
Acrylamide intake, μg/day	21.3 (12.7)	20.9 (11.7)
Coffee, g/day	488 (242)	496 (244)
Dutch spiced cake, g/day	6.0 (9.9)	5.6 (9.3)
Cookies, g/day	14.0 (10.8)	13.8 (10.5)
Potato crisps, g/day	0.38 (1.48)	0.39 (1.87)
French fries, g/day	4.0 (9.4)	3.7 (8.1)
Total energy intake, kcal	1671 (420)	1691 (399)
*Non-dietary variables*		
Age, yrs	61.3 (4.2)	61.5 (4.3)
BMI, kg/m^2^	26.4 (4.2)	25.1 (3.6)
Age at menarche, yrs	13.4	13.7
Age at menopause, yrs	50.1	49.1
Parity, n children	2.2	2.8
n cigarettes per day	3.8 (7.0)	4.5 (7.6)
n smoking years	9.2 (14.5)	11.2 (15.7)
*Cigarette smoking status %*		
Never smokers	64.3	58.9
Former smokers	19.5	20.7
Current smokers	16.2	20.4
Ever use of postmenopausal hormone treatment, % yes	14.5	12.3
Ever use of oral contraceptives, % yes	13.9	24.8
Family history of endometrial cancer, % yes	4.1	3.0

^*^n represents number of subcohort members or cases after exclusion of participants with prevalent cancer at baseline, hysterectomy, incomplete or inconsistent dietary data, and a sample call rate <95%. The number of missing values varies for the variables in this Table.

**Table 2:** Main associations between acrylamide intake and endometrial cancer risk.

Main association acrylamide*								
	N cases	Per 10 μg/day increment	Quintile 1	Quintile 2	Quintile 3	Quintile 4	Quintile 5	P trend
All women, 20.3 yrs FU	393	0.98 (0.88–1.10)	Ref (1.00)	0.87 (0.60–1.27)	0.86 (0.58–1.28)	0.95 (0.64–1.41)	1.03 (0.71–1.51)	0.77
All women, 1^st^ 11.3 yrs FU	221	1.05 (0.92–1.19)	Ref (1.00)	0.98 (0.60–1.59)	1.05 (0.63–1.74)	1.35 (0.82–2.22)	1.36 (0.84–2.19)	0.10
Never-smokers, 20.3 yrs FU	260	1.03 (0.90–1.18)	Ref (1.00)	1.07 (0.67–1.70)	1.14 (0.70–1.86)	1.08 (0.66–1.77)	1.44 (0.90–2.28)	0.17
Never-smokers, 1^st^ 11.3 yrs FU	150	1.13 (0.96–1.33)	Ref (1.00)	1.24 (0.66–2.31)	1.62 (0.87–3.03)	1.56 (0.83–2.92)	2.14 (1.20–3.82)	0.01

^*^Adjusted for age (yrs), age at menarche (yrs), age at menopause (yrs), parity (n children), ever use of oral contraceptives (yes/no), ever use of postmenopausal hormone use (yes/no), BMI (kg/m^2^), (kcal/day) and in the analyses for all women: current smoking (yes/no), quantity of smoking (cigarettes/day), duration of smoking (n smoking years), family history of endometrial cancer (yes/no), energy intake (kcal/day).

**Table 3:** Genetic variants showing a (borderline) statistically significant association with endometrial cancer risk, 11.3 years of follow-up.

Main association SNPs†									Benjamini-Hochberg p value
SNP	Total n cases	1 or 2 variant alleles vs homozygous wild type		1 variant allele vs homozygous wild type		2 variant alleles vs homozygous wild type		P trend per allele	
		n cases	HR (95% CI)	n cases	HR (95% CI)	n cases	HR (95% CI)		
*CYP1A2*, rs2472299	205	115	1.35(1.01–1.81)	94	1.33(0.98–1.81)	21	1.44(0.86–2.40)	0.05	0.60
*CYP1B1*, rs1056827	203	86	0.86(0.64–1.16)	79	0.94(0.69–1.28)	7	0.44(0.20–0.98)	0.09	0.62
*MUTYH*, rs3219489	205	98	1.27(0.95–1.70)	82	1.24(0.92–1.69)	16	1.42(0.81–2.51)	0.09	0.62
*NOS2*, rs944722	198	115	0.81(0.60–1.10)	92	0.89(0.65–1.22)	23	0.61(0.37–0.99)	0.05	0.60
*PGR*, rs660149	205	113	1.45(1.08–1.95)	101	1.53(1.13–2.07)	12	1.04(0.55–1.97)	0.05	0.60
*SULT1A1*, rs1042157	205	141	1.31(0.96–1.80)	105	1.27(0.92–1.77)	36	1.44(0.93–2.23)	0.07	0.60
*SULT1A1*, rs6839	205	127	1.29(0.96–1.74)	93	1.24(0.90–1.71)	34	1.44(0.94–2.22)	0.07	0.60
*XPC*, rs2228000	205	81	0.78(0.58–1.05)	70	0.81(0.60–1.11)	11	0.61(0.32–1.16)	0.06	0.60
*GSTM1* deletion									
	**Total n cases**	**Homozygous deleted vs 1 or 2 copies**		**P for HR**	**Benjamini-Hochberg p value**				
		**n cases**	**HR (95% CI)**						
Deletion based on both *GSTM1* SNPs	205	55	0.80(0.58–1.11)						
Deletion based on rs10857795	205	64	0.80(0.59–1.10)						
Deletion based on rs200184852	205	72	0.71(0.52–0.96)	0.03‡	0.60				

^†^Adjusted for age.

^‡^P value for GSTM1 deletion as assessed by missing call in rs200184852.

**Table 4:** Interactions between SNPs in genes in acrylamide metabolism and dietary acrylamide intake on the risk of endometrial cancer, 11.3 years of follow-up.

Gene, SNP*	Acrylamide, continuous intake	Acrylamide, tertiles of intake							P for interaction	
		Tertile 1		Tertile 2		Tertile 3		P for trend		
	10 μg/day	N cases	HR (95% CI)	N cases	HR (95% CI)	N cases	HR (95% CI)		Raw p	Benjamini-Hochberg p value
All women										
* CYP2E1*, rs915906 = 0	1.17 (1.01–1.35)	31	Ref (1.00)	38	1.28 (0.74–2.20)	58	1.90 (1.15–3.12)	0.01	0.02	0.83
* CYP2E1*, rs915906 = 1	0.75 (0.50–1.12)	21	Ref (1.00)	18	1.07 (0.49–2.34)	12	0.73 (0.30–1.76)	0.49		
Never-smokers										
* CYP2E1*, rs915906 = 0	1.34 (1.09–1.63)	21	Ref (1.00)	25	1.40 (0.71–2.75)	41	2.31 (1.26–4.21)	0.006	0.07	0.61
* CYP2E1*, rs915906 = 1	0.91 (0.55–1.49)	11	Ref (1.00)	13	1.70 (0.65–4.43)	9	1.21 (0.41–3.56)	0.74		
All women										
* CYP2E1*, rs2480258 = 0	1.22 (1.02–1.45)	28	Ref (1.00)	31	1.31 (0.72–2.37)	46	1.82 (1.06–3.11)	0.03	0.03	0.83
* CYP2E1*, rs2480258 = 1	0.88 (0.69–1.11)	24	Ref (1.00)	25	1.09 (0.56–2.12)	24	1.13 (0.57–2.23)	0.74		
Never-smokers										
* CYP2E1*, rs2480258 = 0	1.37 (1.10–1.72)	20	Ref (1.00)	21	1.44 (0.70–2.96)	34	2.24 (1.19–4.20)	0.01	0.11	0.70
* CYP2E1*, rs2480258 = 1	0.96 (0.71–1.31)	12	Ref (1.00)	17	1.70 (0.76–3.83)	16	1.56 (0.65–3.74)	0.34		
All women										
* GSTM1* 1 or 2 copies, all SNPs	1.12 (0.97–1.30)	36	Ref (1.00)	36	1.04 (0.60–1.81)	59	1.66 (1.00–2.74)	0.04	0.14†	0.92
* GSTM1* deleted, all SNPs	0.90 (0.63–1.30)	16	Ref (1.00)	20	1.68 (0.71–3.99)	11	0.93 (0.39–2.21)	0.94		
Never-smokers										
* GSTM1* 1 or 2 copies, all SNPs	1.25 (1.05–1.49)	21	Ref (1.00)	24	1.52 (0.75–3.05)	42	2.56 (1.39–4.68)	0.002	0.28	0.86
* GSTM1* deleted, all SNPs	0.90 (0.53–1.54)	11	Ref (1.00)	14	1.78 (0.67–4.72)	8	0.78 (0.27–2.23)	0.73		
All women										
* GSTT1* 1 or 2 copies, all SNPs	1.13 (0.98–1.30)	48	Ref (1.00)	52	1.23 (0.78–1.93)	66	1.60 (1.04–2.44)	0.03	0.07	0.92
* GSTT1* deleted, all SNPs	0.55 (0.23–1.28)	4	Ref (1.00)	4	0.83 (0.13–5.41)	4	0.28 (0.03–2.77)	0.24		
Never-smokers										
* GSTT1* 1 or 2 copies, all SNPs	1.33 (1.10–1.61)	29	Ref (1.00)	35	1.56 (0.88–2.76)	49	2.35 (1.39–3.98)	0.001	0.02	0.61
* GSTT1* deleted, all SNPs	0.43 (0.19–0.97)	3	Ref (1.00)	3	0.83 (0.17–4.10)	1	0.13 (0.01–2.05)	0.08		

Adjusted for age (yrs), age at menarche (yrs), age at menopause (yrs), parity (n children), ever use of oral contraceptives (yes/no), ever use of postmenopausal hormone use (yes/no), BMI (kg/m^2^), current smoking (yes/no), quantity of smoking (cigarettes/day), duration of smoking (n smoking years), family history of endometrial cancer (yes/no),energy intake (kcal/day).

^*^0 = homozygous wild type, 1 = 1 or 2 variant alleles.

^†^p for interaction borderline statistically significant (p = 0.09) when deletion was based on missing calls for rs200184852.
